# Tree inhabiting gnomoniaceous species from China, with *Cryphogonomonia* gen. nov. proposed

**DOI:** 10.3897/mycokeys.69.54012

**Published:** 2020-07-10

**Authors:** Qin Yang, Ning Jiang, Cheng-Ming Tian

**Affiliations:** 1 Forestry Biotechnology Hunan Key Laboratories, Central South University of Forestry and Technology, Changsha 410004, China Beijing Forestry University Beijing China; 2 The Key Laboratory for Silviculture and Conservation of the Ministry of Education, Beijing Forestry University, Beijing 100083, China Central South University of Forestry and Technology Changsha China

**Keywords:** forest trees, Gnomoniaceae, new genus, phylogeny, systematics

## Abstract

Species of Gnomoniaceae are commonly associated with leaf spot diseases of a wide range of plant hosts worldwide. During our investigation of fungi associated with tree diseases in China, several gnomoniaceous isolates were recovered from symptomatic branches and leaves on different woody plants in the Fagaceae, Pinaceae, and Salicaceae families. These isolates were studied by applying a polyphasic approach including morphological, cultural data, and phylogenetic analyses of partial ITS, LSU, *tef1*, *rpb2* and *tub2* gene sequences. As a result, three species were identified with characters fitting into the family Gnomoniaceae. One of these species is described herein as *Cryphognomonia pini***gen. et sp. nov.**, characterized by developed pseudostromata and ascospores with obvious hyaline sheath; *Gnomoniopsis xunwuensis***sp. nov.** is illustrated showing sympodially branched conidiophore, oval or fusiform conidia; and one known species, *Plagiostoma populinum*. The current study improves the understanding of gnomoniaceous species causing diebacks and leaf spot on ecological and economic forest trees.

## Introduction

The Gnomoniaceae (Diaporthales, Sordariomycetes, Ascomycota) is a family of perithecial ascomycetes that occur as endophytes, pathogens, or saprobes on growing and overwintered leaves of hardwood trees, shrubs, and herbaceous plants (Walker 2012). Many species in the Gnomoniaceae cause serious tree diseases such as cherry leaf scorch (*Apiognomonia erythrostoma* (Pers.) Höhn.), oak dieback (*A. errabunda* (Roberge) Höhn), sycamore canker (*A. veneta* (Sacc. & Speg.) Höhn), and chestnut dieback (*Gnomoniopsis daii* Tian & Jiang) (Sogonov et al. 2008; Walker et al. 2010; Jiang et al. 2019).

The sexual morph of Gnomoniaceae is characterized by ascomata that are generally immersed, solitary or aggregated in an undeveloped stroma (Rossman et al. 2007; Sogonov et al. 2008). The perithecia are dark brown to black and pseudoparenchymatous with central, eccentric, or lateral necks (Rossman et al. 2007; Sogonov et al. 2008). Asci usually have an inconspicuous or distinct apical ring. Ascospores are generally small, hyaline, uniseptate. The asexual morph is characterized by acervular or pycnidial, phialidic, with non-septate conidia (Monod 1983).

The generic concepts of Gnomoniaceae were recently revised based on a survey of leaf-inhabiting diaporthalean fungi (Sogonov et al. 2008). Phylogenetic analyses of molecular markers is the primary methodology for systematic studies of the Gnomoniaceae, however, host specificity and morphology can also be useful for species identification. Recent phylogenetic studies have shown that species of Gnomoniaceae often have a narrow host range associating with a single host genus or species (Mejía et al. 2008, 2011a, b, 2012; Sogonov et al. 2008; Walker et al. 2010, 2012, 2013). For example, *Cryptosporella* is a well-defined genus which was frequently limited to a single host species, especially in the host family Betulaceae, except for *C. wehmeyeriana* on *Tilia* spp. and type species *C. hypodermia* on *Ulmus* spp. (Mejía et al. 2008, 2011b).

Several fungal species of Gnomoniaceae, *Cryptosporella platyphylla* from *Betula platyphylla*, *Flavignomonia rhoigena* from *Rhus chinensis*, *Gnomoniopsis daii* and *Ophiognomonia castaneae* from *Castanea mollissima*, have been reported from China (Fan et al. 2016; Gong et al. 2017; Jiang and Tian 2019; Jiang et al. 2019). In the present study, tree inhabiting gnomoniaceous species, mainly on cankered branches and leaves, were surveyed in China. The aim of the present study was to identify these fungi via morphology and multi-locus phylogeny based on modern taxonomic concepts.

## Materials and methods

### Isolates

Fresh specimens of Gnomoniaceae-related fungi were collected from branches and leaves of hosts in Beijing, Jiangxi and Shaanxi provinces (Tables 1–3). Isolates from host material were obtained by removing a mucoid spores mass from perithecia and pycnidia-like conidiomata, spreading the suspension on the surface of 1.8% potato dextrose agar (PDA), and incubating at 25 °C for up to 24 h. Single germinating conidia/ascospore was removed and plated on to fresh PDA plates. Specimens are deposited in the Museum of the Beijing Forestry University (**BJFC**). Axenic cultures are maintained in the China Forestry Culture Collection Centre (**CFCC**).

### Morphological analysis

Morphological observations of the asexual/sexual morph in the natural environment were based on features of the conidiomata or ascomata on infected plant tissues and micromorphology, supplemented by cultural characteristics. Ascomata and conidiomata from tree barks were sectioned by hand, using a double-edged blade and structures were observed under a dissecting microscope. The gross morphology of conidiomata or ascomata was recorded using a Leica stereomicroscope (M205 FA). Fungal structures were mounted in clear lactic acid and micromorphological characteristics were examined using a Leica compound microscope (DM 2500) with differential interference contrast (DIC) optics. Thirty measurements of each structure were determined for each collection. Colony characters and pigment production on PDA were noted after 10 d. Colony colors were described according to Rayner (1970).

### DNA extraction, PCR amplification and sequencing

Total genomic DNA was extracted from fresh mycelium grown on PDA using a cetyltrimethylammonium bromide (CTAB) method (Doyle and Doyle 1990). PCR amplifications were performed in a DNA Engine Peltier Thermal Cycler (PTC-200; Bio-Rad Laboratories, Hercules, CA, USA). The primer sets ITS1 and ITS4 (White et al. 1990) were used to amplify the ITS region. The primer sets LR0R and LR7 (Vilgalys and Hester 1990; Vilgalys and Sun 1994) were used to amplify the nuclear ribosomal large subunit (LSU) region. The primer sets EF1-728F (Carbone and Kohn 1999) and EF1-1567R (Rehner 2001) were used to amplify a partial fragment of the translation elongation factor 1-α gene (tef1-α). The primer sets RPB2-5F and fRPB2-7cR (Liu et al. 1999) were used to amplify the partial RNA polymerase II subunit (rpb2) region. The primer sets T1 (O’Donnell and Cigelnik 1997) and Bt2b (Glass and Donaldson 1995) were used to amplify the beta-tubulin gene (tub2). The PCR conditions were: an initial denaturation step of 5 min at 94 °C followed by 35 cycles of 30 sec at 94 °C, 50 sec at 48 °C (ITS, LSU) or 54 °C (*tef-1*α) or 55 °C (*rpb2*, *tub2*) and 1 min at 72 °C, and a final elongation step of 7 min at 72 °C. PCR amplification products were assayed via electrophoresis in 2% agarose gels. DNA sequencing was performed using an ABI PRISM 3730XL DNA Analyser with a BigDye Terminater Kit v.3.1 (Inv-itrogen, USA) at the Shanghai Invitrogen Biological Technology Company Limited (Beijing, China).

### Phylogenetic analyses

The quality of our amplified nucleotide sequences was checked and combined by SeqMan v.7.1.0 and reference sequences were retrieved from the National Center for Biotechnology Information (NCBI), based on Mejía et al. (2011a), Senanayake et al. (2018), Jiang and Tian (2019), and Jiang et al. (2019), supplemented by sequences of *Tenuignomonia styracis* and *Neognomoniopsis quercina* from Crous et al. (2019) and Minoshima et al. (2019). Sequences were aligned using MAFFT v. 6 (Katoh and Toh 2010) and manually corrected using Bioedit 7.0.9.0 (Hall 1999).

The phylogenetical analyses were conducted using Maximum Parsimony (MP), Maximum Likelihood (ML) and Bayesian inference (BI). MP was performed with PAUP v. 4.0b10 (Swofford 2003) using tree-bisection-reconnection (TBR) as the branch-swapping algorithm. Other calculated parsimony scores were tree length (TL), consistency index (CI), retention index (RI), and rescaled consistency (RC). ML was performed with RAxML (Stamatakis 2006) as implemented in raxmlGUI 1.3 (Silvestro and Michalak 2012), using the ML + rapid bootstrap setting and the GTRGAMMA substitution model with 1000 bootstrap replicates. BI was performed using a Markov Chain Monte Carlo (MCMC) algorithm in MrBayes v. 3.0b4 (Ronquist and Huelsenbeck 2003). Two MCMC chains, started from random trees for 1,000,000 generations and trees, were sampled every 100^th^ generation, resulting in a total of 10,000 trees. The first 25% of trees were discarded as burn-in of each analysis. Branches with significant Bayesian Posterior Probabilities (BPP) were estimated in the remaining 7500 trees. Phylogenetic trees were viewed with FigTree v.1.4.3 (Rambaut 2016) and processed by Adobe Illustrator CS5. Alignment and trees were deposited in TreeBASE (submission ID: S26271). The nucleotide sequence data of the new taxa have been deposited in GenBank (Tables 1–3).

## Results

### Phylogenetic analyses

**Figure 1.**
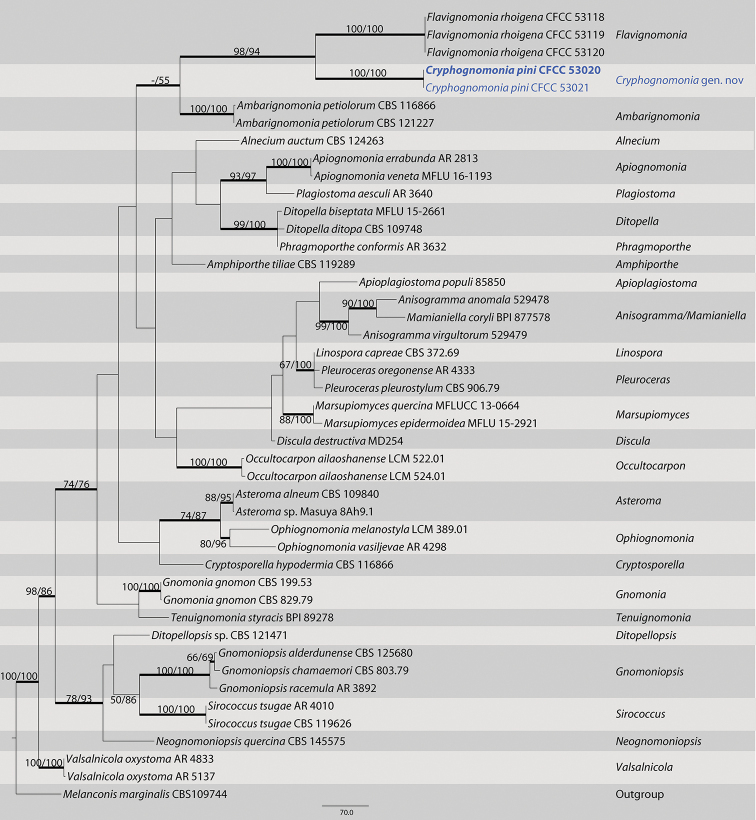
Maximum parsimony phylogram of Gnomoniaceae based on a combined matrix of ITS, LSU, *tef1* and *rpb2* genes. The MP and ML bootstrap support values above 50% are shown at the first and second position, respectively. Thickened branches represent posterior probabilities above 0.90 from BI. Scale bar: 80 nucleotide substitutions. Strains in this study are in blue and ex-type strains are in blod.

The first sequences dataset for the ITS, LSU, *tef1*, and *rpb2* was analyzed to focus on Gnomoniaceae. The alignment included 45 taxa, including the outgroup sequences of *Melanconis marginalis* (Table 1). The aligned four-locus datasets included 3388 characters. Of these, 2180 characters were constant, 198 variable characters were par-simony-uninformative and 1010 characters were parsimony informative. The heuristic search using maximum parsimony (MP) generated 4 parsimonious trees (TL = 3241, CI = 0.539, RI = 0.672, RC = 0.362), from which one was selected (Fig. 1). In the phylogenetic tree, two strains form a well-supported clade (MP/ML/BI=100/100/1) sister to the species *Flavignomonia rhoigena* from *Rhus chinensis*.

**Table 1.** Strains and GenBank accession numbers used in the phylogenetic analyses of Gnomoniaceae

Species	Strains	Genbank accession number			
		ITS	LSU	*tef1*	*rpb2*
*Alnecium auctum*	CBS 124263	KF570154	KF570154	KF570200	KF570170
*Ambarignomonia petiolorum*	CBS 116866	EU199193	AY818963	NA	EU199151
	CBS 121227	EU254748	EU255070	EU221898	EU219307
*Amphiporthe tiliae*	CBS 119289	EU199178	EU199122	NA	EU199137
*Anisogramma anomala*	529478	EU683064	EU683066	NA	NA
*Anisogramma virgultorum*	529479	EU683062	EU683065	NA	NA
*Apiognomonia errabunda*	AR 2813	DQ313525	NG027592	DQ313565	DQ862014
*Apiognomonia veneta*	MFLUCC 16-1193	MF190114	MF190056	NA	NA
*Apioplagiostoma populi*	858501	KP637024	NA	NA	NA
*Asteroma alneum*	CBS 109840	EU167609	EU167609	NA	NA
*Asteroma* sp.	Masuya 8Ah9-1	NA	AB669035	NA	NA
***Cryphognomonia pini***	**CFCC 53020**	**MK432672**	**MK429915**	**MK578144**	**MK578100**
	**CFCC 53021**	**MK432673**	**MK429916**	**MK578145**	**MK578101**
*Cryptosporella hypodermia*	CBS 116866	EU199181	AF408346	NA	EU199140
*Discula destructiva*	MD 254	AF429741	AF429721	AF429732	NA
*Ditopella biseptata*	MFLU 15-2661	MF190147	MF190091	NA	MF377616
*Ditopella ditopa*	CBS 109748	DQ323526	EU199126	NA	EU199145
*Ditopellopsis* sp.	CBS 121471	EU254763	EU255088	EU221936	EU219254
*Flavignomonia rhoigena*	CFCC 53118	MK432674	MK429917	NA	MK578102
	CFCC 53119	MK432675	MK429918	NA	MK578103
	CFCC 53120	MK432676	MK429919	NA	MK578104
*Gnomonia gnomon*	CBS 199.53	DQ491518	AF408361	EU221885	EU219295
	CBS 829.79	AY818957	AY818964	EU221905	NA
*Gnomoniopsis alderdunensis*	CBS 125680	GU320825	NA	NA	NA
*Gnomoniopsis chamaemori*	CBS 803.79	EU254808	EU255107	NA	NA
*Gnomoniopsis racemula*	AR 3892	EU254841	EU255122	EU221889	EU219241
*Mamianiella coryli*	BPI 877578	EU254862	NA	NA	NA
*Marsupiomyces quercina*	MFLUCC 13-0664	MF190116	MF190061	NA	NA
*Marsupiomyces epidermoidea*	MFLU 15-2921	NA	MF190058	NA	NA
*Melanconis marginalis*	CBS 109744	EU199197	AF408373	EU221991	EU219301
*Neognomoniopsis quercina*	CBS 145575	MK876399	MK876440	NA	NA
*Occultocarpon ailaoshanense*	LCM 524.01	JF779849	JF779853	NA	JF779856
	LCM 522.01	JF779848	JF779852	JF779862	JF779857
*Ophiognomonia melanostyla*	LCM 389.01	JF779850	JF779854	NA	JF779858
*Ophiognomonia vasiljevae*	AR 4298	EU254977	EU255162	EU221999	EU219331
*Plagiostoma aesculi*	AR 3640	EU254994	EU255164	NA	EU219269
*Linospora capreae*	CBS 372.69	NA	AF277143	NA	NA
*Pleuroceras oregonense*	AR 4333	EU255060	EU255196	EU221931	EU219313
*Pleuroceras pleurostylum*	CBS 906.79	EU255061	EU255197	EU221962	EU219311
*Phragmoporthe conformis*	AR 3632	NA	AF408377	NA	NA
*Valsalnicola oxystoma*	AR 5137	JX519561	NA	NA	NA
	AR 4833	JX519559	JX519563	NA	NA
*Sirococcus tsugae*	AR 4010	EF512478	EU255207	EU221928	EU219289
	CBS 119626	EU199203	EU199136	EF512534	EU199159
*Tenuignomonia styracis*	BPI 89278	NA	LC379288	LC379282	LC379294

Note: NA, not applicable. Strains in this study are marked in bold.

**Figure 2.**
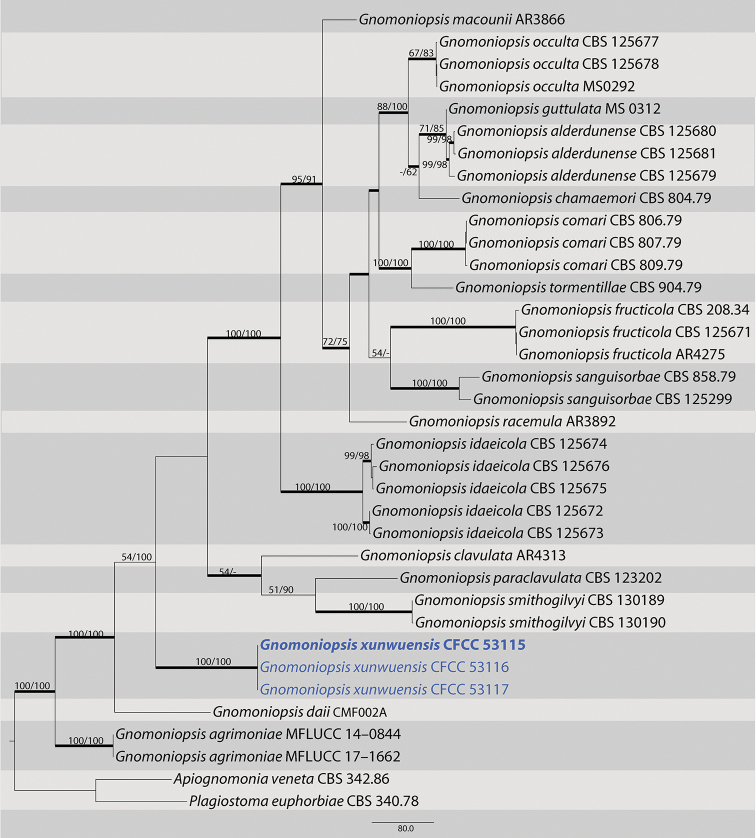
Maximum parsimony phylogram of *Gnomoniosis* based on a combined matrix of ITS, *tef1*-α and *tub2* genes. The MP and ML bootstrap support values above 50% are shown at the first and second position, respectively. Thickened branches represent posterior probabilities above 0.90 from BI. Scale bar: 80 nucleotide substitutions. Strains in this study are in blue and ex-type strains are in blod.

The second dataset with ITS, *tef1* and *tub2* sequences were analyzed in combination to infer the interspecific relationships within *Gnomoniopsis*. The alignment included 36 taxa, including the outgroup sequences of *Apiognomonia veneta* and *Plagiostoma euphorbiae* (Table 2). The aligned three-locus datasets included 2481 characters. Of these, 1443 characters were constant, 186 variable characters were par-simony-uninformative and 852 characters were parsimony informative. The heuristic search using maximum parsimony (MP) generated one parsimonious tree (TL = 2644, CI = 0.620, RI = 0.781, RC = 0.485), which is shown in Fig. 2. In the phylogenetic tree, three strains form a well-supported clade (MP/ML/BI=100/100/1) that does not include any previously described species.

**Table 2.** Strains and GenBank accession numbers used in the phylogenetic analyses of *Gnomoniopsis*

Species	Strain	Genbank accession number		
		ITS	*tef1*	*tub2*
*Apiognomonia veneta*	CBS 342.86	DQ313531	DQ318036	EU219235
*Gnomoniopsis alderdunensis*	CBS 125679	GU320826	GU320813	GU320788
	CBS 125680	GU320825	GU320801	GU320787
	CBS 125681	GU320827	GU320802	GU320789
*Gnomoniopsis chamaemori*	CBS 804.79	GU320817	GU320809	GU320777
*Gnomoniopsis chinensis*	CFCC 52286	MG866032	MH545370	MH545366
	CFCC 52287	MG866033	MH545371	MH545367
	CFCC 52288	MG866034	MH545372	MH545368
	CFCC 52289	MG866035	MH545373	MH545369
*Gnomoniopsis clavulata*	CBS 121255	EU254818	GU320807	EU219211
*Gnomoniopsis comari*	CBS 806.79	EU254821	GU320810	EU219156
	CBS 807.79	EU254822	GU320814	GU320779
	CBS 809.79	EU254823	GU320794	GU320778
*Gnomoniopsis daii*	CFCC 54043	MN598671	MN605519	MN605517
	CMF002B	MN598672	MN605520	MN605518
*Gnomoniopsis fructicola*	CBS 121226	EU254824	GU320792	EU219144
	CBS 208.34	EU254826	GU320808	EU219149
	CBS 125671	GU320816	GU320793	GU320776
*Gnomoniopsis guttulata*	MS 0312	EU254812	NA	NA
*Gnomoniopsis idaeicola*	CBS 125672	GU320823	GU320797	GU320781
	CBS 125673	GU320824	GU320798	GU320782
	CBS 125674	GU320820	GU320796	GU320780
	CBS 125675	GU320822	GU320799	GU320783
	CBS 125676	GU320821	GU320811	GU320784
*Gnomoniopsis macounii*	CBS 121468	EU254762	GU320804	EU219126
*Gnomoniopsis occulta*	CBS 125677	GU320828	GU320812	GU320785
	CBS 125678	GU320829	GU320800	GU320786
*Gnomoniopsis paraclavulata*	CBS 123202	GU320830	GU320815	GU320775
*Gnomoniopsis racemula*	CBS 121469	EU254841	GU320803	EU219125
*Gnomoniopsis sanguisorbae*	CBS 858.79	GU320818	GU320805	GU320790
*Gnomoniopsis smithogilvyi*	CBS 130190	JQ910642	KR072534	JQ910639
	CBS 130189	JQ910644	KR072535	JQ910641
	CBS 130188	JQ910643	KR072536	JQ910640
	MUT 401	HM142946	KR072537	KR072532
	MUT 411	HM142948	KR072538	KR072533
*Gnomoniopsis tormentillae*	CBS 904.79	EU254856	GU320795	EU219165
***Gnomoniopsis xunwuensis***	**CFCC 53115**	**MK432667**	**MK578067**	**MK578141**
	**CFCC 53116**	**MK432668**	**MK578068**	**MK578142**
	**CFCC 53117**	**MK432669**	**MK578069**	**MK578143**
*Plagiostoma euphorbiae*	CBS 340.78	DQ323532	GU354016	GU367034

Note: NA, not applicable. Strains in this study are marked in bold.

**Figure 3.**
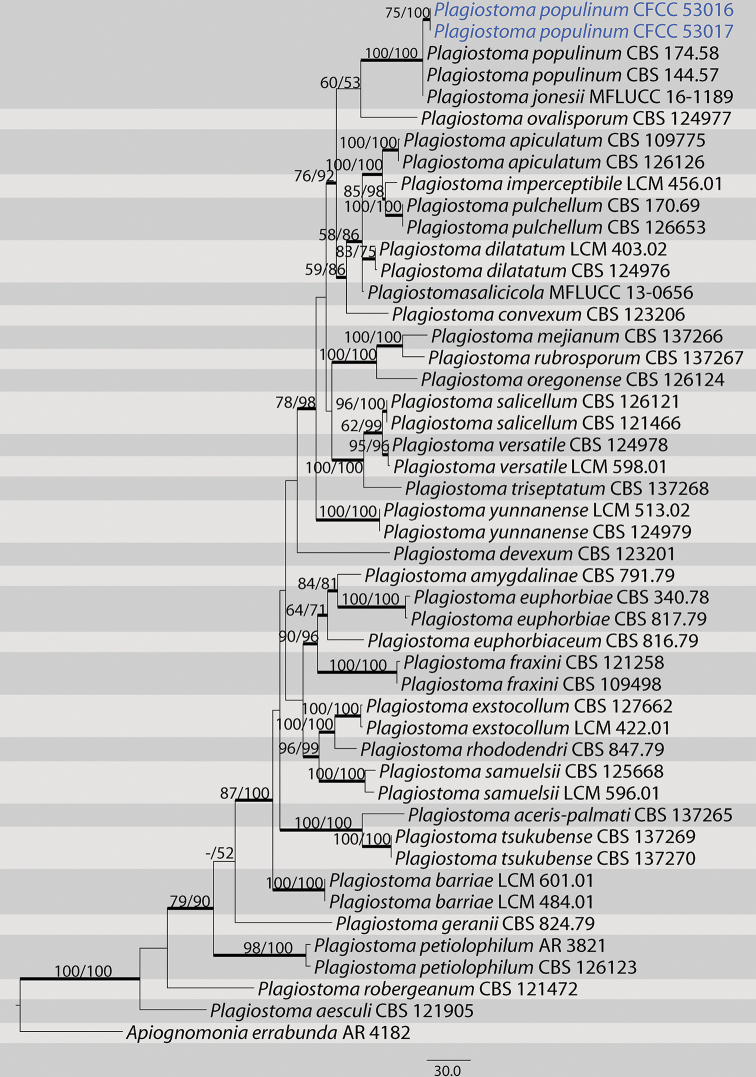
Maximum parsimony phylogram of *Plagiostoma* based on a combined matrix of ITS, *tef1*-α and *tub2* genes. The MP and ML bootstrap support values above 50% are shown at the first and second position, respectively. Thickened branches represent posterior probabilities above 0.90 from BI. Scale bar: 30 nucleotide substitutions. Strains in this study are in blue.

The third dataset with ITS, *tef1* and *tub2* sequences were analyzed in combination to infer the interspecific relationships within *Plagiostoma*. The alignment included 48 taxa, including the outgroup sequences of *Apiognomonia errabunda* (Table 3). The aligned three-locus datasets included 2311 characters. Of these, 1556 characters were constant, 204 variable characters were parsimony-uninformative and 551 characters were parsimony informative. The heuristic search using maximum parsimony (MP) generated 6 parsimonious trees (TL = 1462, CI = 0.685, RI = 0.779, RC = 0.534), from which one was selected (Fig. 3). In the phylogenetic tree, four strains from this study group in a well-supported clade with *Plagiostoma populinum*. The topologies resulting from MP, ML and BI analyses of the concatenated dataset were congruent.

**Table 3.** Strains and GenBank accession numbers used in the phylogenetic analyses of *Gnomoniopsis*.

**Species**	**Strain**	**Genbank accession number**		
		**ITS**	***tef1***	***tub2***
*Apiognomonia errabunda*	AR 4182	DQ313543	KJ509937	KJ509947
*Plagiostoma aceris-palmati*	CBS 137265	KJ509959	KJ509938	KJ509949
*Plagiostoma aesculi*	CBS 121905	EU254994	GU367022	GU354005
*Plagiostoma amygdalinae*	CBS 791.79	EU254995	GU367030	GU354012
*Plagiostoma apiculatum*	CBS 109775	DQ323529	GU367008	GU353990
	CBS 126126	GU367066	GU367009	GU353991
*Plagiostoma barriae*	LCM 601.01	GU367054	GU366997	GU353980
	LCM 484.01	GU367053	GU366995	GU353979
*Plagiostoma convexum*	CBS 123206	EU255047	EU219112	GU353994
*Plagiostoma devexum*	CBS 123201	EU255001	GU367027	GU354010
*Plagiostoma dilatatum*	LCM 403.02	GU367069	GU367012	GU353995
	CBS 124976	GU367070	GU367014	GU353996
*Plagiostoma euphorbiaceum*	CBS 816.79	EU255003	EU219158	GU354013
*Plagiostoma euphorbiae*	CBS 340.78	DQ323532	GU367034	GU354016
	CBS 817.79	KJ509960	GU367028	KJ509950
*Plagiostoma exstocollum*	CBS 127662	GU367046	GU366988	GU353972
	LCM 422.01	GU367043	GU366989	GU353969
*Plagiostoma fraxini*	CBS 121258	EU255008	KJ509939	KJ509951
	CBS 109498	AY455810	GU367033	GU354015
*Plagiostoma geranii*	CBS 824.79	EU255009	GU367032	GU354014
*Plagiostoma imperceptibile*	LCM 456.01	GU367059	GU367002	GU353984
*Plagiostoma jonesii*	MFLUCC 16–1189	MF190159	NA	MF377589
*Plagiostoma mejianum*	CBS 137266	KJ509961	KJ509940	KJ509952
*Plagiostoma oregonense*	CBS 126124	GU367073	GU367016	GU353999
*Plagiostoma ovalisporum*	CBS 124977	GU367072	GU367015	GU353998
*Plagiostoma petiolophilum*	AR 3821	EU255039	GU367025	GU354008
	CBS 126123	GU367078	GU367023	GU354006
***Plagiostoma populinum***	**CFCC 53016**	**MK432677**	**MK578070**	**MK578146**
	**CFCC 53017**	**MK432678**	**MK578071**	**MK578147**
*Plagiostoma populinum*	CBS 174.58	GU367074	GU367017	GU354000
	CBS 144.57	GU367075	GU367018	GU354001
*Plagiostoma pulchellum*	CBS 170.69	EU255043	KJ509941	GU353989
	CBS 126653	GU367063	GU367006	GU353987
*Plagiostoma rhododendri*	CBS 847.79	EU255044	GU367026	GU354009
*Plagiostoma robergeanum*	CBS 121472	EU255046	GU367029	GU354011
*Plagiostoma rubrosporum*	CBS 137267	KJ509962	KJ509942	KJ509953
*Plagiostoma salicellum*	CBS 126121	GU367037	GU366977	GU353961
	CBS 121466	EU254996	GU366978	GU353962
*Plagiostoma salicicola*	MFLUCC 13–0656	MF190161	NA	NA
*Plagiostoma samuelsii*	CBS 125668	GU367051	GU366993	GU353977
	LCM 596.01	GU367052	GU366994	GU353978
*Plagiostoma triseptatum*	CBS 137268	KJ509963	KJ509943	KJ509954
*Plagiostoma tsukubense*	CBS 137269	KJ509964	KJ509944	KJ509955
	CBS 137270	KJ509965	KJ509945	KJ509956
*Plagiostoma versatile*	CBS 124978	GU367038	GU366979	GU393963
	LCM 598.01	GU367040	GU366981	GU393965
*Plagiostoma yunnanense*	LCM 513.02	GU367036	GU366976	GU353960
	CBS 124979	GU367035	GU366975	GU353959

Note: NA, not applicable. Strains in this study are marked in bold.

## Taxonomy

### Cryphognomonia

Taxon classificationFungiDiaporthalesGnomoniaceae

C.M. Tian & N. Jiang gen. nov.

C9CCE66F-0558-5DBF-BB0E-433C484F1A90

829509

#### Etymology.

*Crypho* + *gnomonia*, referring to the cryptic stromata on hosts.

#### Type species.

*Cryphognomonia pini* C.M. Tian & N. Jiang

#### Description.

***Pseudostromata*** erumpent, causing a pustulate bark surface. ***Central column*** yellowish to brownish. ***Stromatic zones*** lacking. ***Perithecia*** conspicuous, flask-shaped to spherical, umber to fuscous black, regularly scattered. ***Paraphyses*** deliquescent. ***Asci*** fusoid, 8-spored, biseriate, with an apical ring. ***Ascospores*** hyaline, clavate to cylindrical, smooth, multi-guttulate, symmetrical to asymmetrical, straight to slightly curved, bicellular, with a median septum distinctly constricted, with distinct hyaline sheath. ***Asexual morph***: not observed.

#### Notes.

*Cryphognomonia* was classified as a new genus in Gnomoniaceae throughout molecular data and the characteristics of sexual morph. Morphologically, *Cryphognomonia* can be distinguished from the other genera by pseudostromata and ascospores with obvious hyaline sheath.

### Cryphognomonia pini

Taxon classificationFungiDiaporthalesGnomoniaceae

C.M. Tian & N. Jiang sp. nov.

DDB2516A-8F2F-5985-8344-22CAEA479CB2

829510

Figure 4

#### Diagnosis.

*Cryphognomonia pini* differs from its closest phylogenetic neighbor, *F. rhoigena*, in ITS, LSU, *tef1* and *rpb2* loci based on the alignments deposited in TreeBASE.

#### Etymology.

Named after the genus of the host plant from which the holotype was collected, *Pinus*.

#### Description.

***Pseudostromata*** erumpent, causing a pustulate bark surface, 650–1200 µm diam., containing up to 12 perithecia. ***Central column*** yellowish to brownish. ***Stromatic zones*** lacking. *Perithecia* conspicuous, flask-shaped to spherical, umber to fuscous black, regularly scattered, 350–600 µm diam. ***Paraphyses*** deliquescent. ***Asci*** fusoid, 8-spored, biseriate, with an apical ring, (60–)65–80(–90) × (21–)22–31(–35) µm. ***Ascospores*** hyaline, clavate to cylindrical, smooth, multi-guttulate, symmetrical to asymmetrical, straight to slightly curved, bicellular, with a median septum distinctly constricted, with distinct hyaline sheath, (15.5–)18–25(–27) × (8.5–)9.5–11.5(–12) µm. ***Asexual morph***: not observed.

#### Culture characters.

Cultures incubated on PDA at 25 °C in the dark, initially pale white, becoming olive-green after 3 wk. The colonies are flat, with regular margins; texture initially uniform, becoming compact after 1 month.

#### Specimens examined.

China. Shaanxi Province: Ankang City, Huoditang forest farm, 33°26'7"N, 108°26'48"E, on branches of *Pinus armandii*, 8 June 2018, *N. Jiang* & *C.M. Tian* (holotype BJFC-S1725; ex-type living culture: CFCC 53020); 33°26'7"N, 108°26'48"E, on branches of *Pinus armandii*, 8 June 2018, *N. Jiang* & *C.M. Tian* (BJFC-S1726; living culture: CFCC 53021).

#### Notes.

*Cryphognomonia pini* is the type species of *Cryphognomonia*, and occurs on *Pinus armandii* in China. Morphologically, *Cryphognomonia pini* is characterized based on bicellular ascospores with obvious hyaline sheath. In the phylogenetic tree, this species is most closely related to *F. rhoigena* (Fig. 1). However, *Cryphognomonia pini* can be distinguished from *F. rhoigena* based on ITS, LSU, *tef1* and *rpb2* loci (73/512 in ITS, 4/775 in LSU, 186/437 in *tef1* and 90/1064 in *rpb2*).

**Figure 4.**
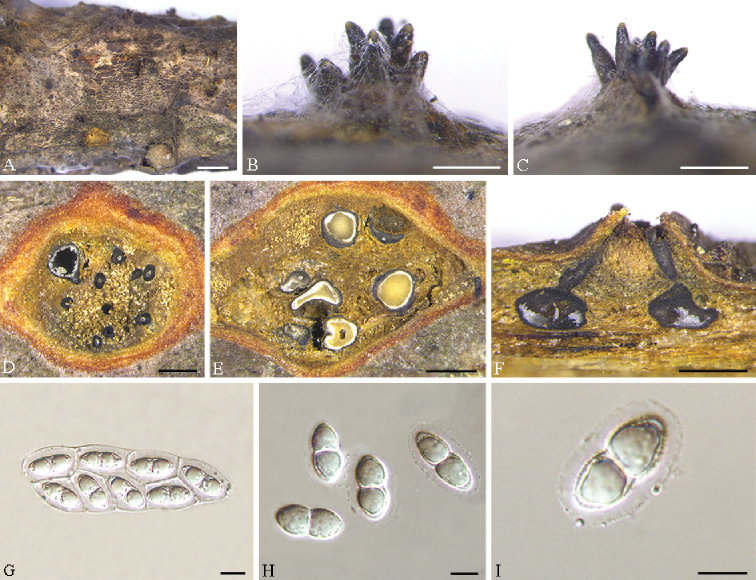
*Cryphognomonia pini* on *Pinus armandii* (BJFC-S1725) **A–C** habit of ascomata on twigs **D, E** transverse section of ascomata **F** longitudinal section through ascomata **G** asci **H, I** ascospores. Scale bars: 2 mm (**A**); 500 μm (**B–F**); 10 μm (**G–I**).

### Gnomoniopsis xunwuensis

Taxon classificationFungiDiaporthalesGnomoniaceae

C.M. Tian & Q. Yang sp. nov.

C1F2771E-F584-598A-B96A-C7DF66F49514

829529

Figure 5

#### Diagnosis.

*Gnomoniopsis xunwuensis* differs from its closest phylogenetic neighbor, *G. daii*, in ITS, *tef1* and *tub2* loci based on the alignments deposited in TreeBASE.

#### Etymology.

Named after the County (Xunwu), where the species was first collected.

**Figure 5.**
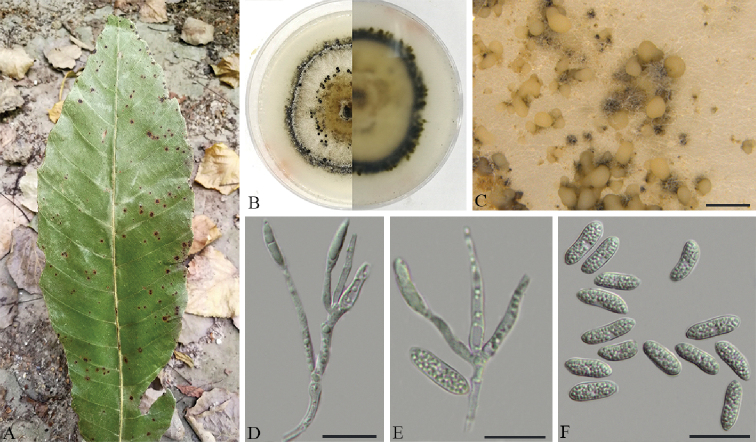
*Gnomoniopsis xunwuensis* on *Castanopsis fissa* (BJFC-S1688) **A** symptoms on leaves of host plant **B** the colony on PDA**C** conidiomata on PDA**D, E** conidiophores attached with condia **F** conidia. Scale bars: 500 μm (**C**); 20 μm (**D–F**).

#### Description.

On PDA: ***Conidiomata*** pycnidial, (115–)130–210(–250) μm diam., globose, solitary to gregarious, or occasionally coalescing, deeply embedded in the medium, erumpent, brown to dark black. White to cream conidial drops exuding from the ostioles. ***Conidiophores*** (40–)43–58(–60.5) × 2–2.5(–3) μm, cylindrical, hyaline, phiailidic, branched or sympodially branched, straight or slightly curved. ***Conidia*** oval or fusiform, straight to slightly curved, hyaline, multiguttules, (14–)16.5–20 × 4–5.5 µm.

#### Culture characters.

Cultures incubated on PDA at 25 °C in the dark. Colony originally compact and flat with white aerial mycelium, then developing pale brown aerial mycelium at the center and blackish green mycelium at the marginal area, zonate with 2 well defined zones with regular edge; conidiomata dense, regularly distributed over agar surface.

#### Specimens examined.

China. Jiangxi Province: Ganzhou City, Xunwu County, 24°40'50"N, 115°34'37"E, on leaves of *Castanopsis fissa*, 12 May 2018, *Q. Yang*, *Y. Liu* & *Y.M. Liang* (holotype BJFC-S1688; ex-type living culture: CFCC 53115); 24°52'20"N, 115°35'25"E, on leaves of *Castanopsis fissa*, 12 May 2018, *Q. Yang*, *Y. Liu* & *Y.M. Liang* (BJFC-S1689; living culture: CFCC 53116 and CFCC 53117).

#### Notes.

*Gnomoniopsis xunwuensis* is associated with leaf spot of *Castanopsis fissa*, representing the first report from this host in China. It is characterized by sympodially branched conidiophore and oval or fusiform conidia. Morphologically, *G. xunwuensis* differs from *G. daii* in having bigger conidia (16.5–20 × 4–5.5 vs. 5.5–7 × 2–3.5 µm) (Jiang and Tian 2019). The phylogenetic inferences indicated this species as an individual well-supported clade (MP/ML/BI=100/100/1) in the genus *Gnomoniopsis* (Fig. 2).

### Plagiostoma populinum

Taxon classificationFungiDiaporthalesGnomoniaceae

(Fuckel) L.C. Mejía. Stud. Mycol. 68: 225. 2011.

ACBB0FE2-EF21-5D11-B50D-8F58DF82058D

Figure 6

#### Description.

See Butin (1958)

**Figure 6.**
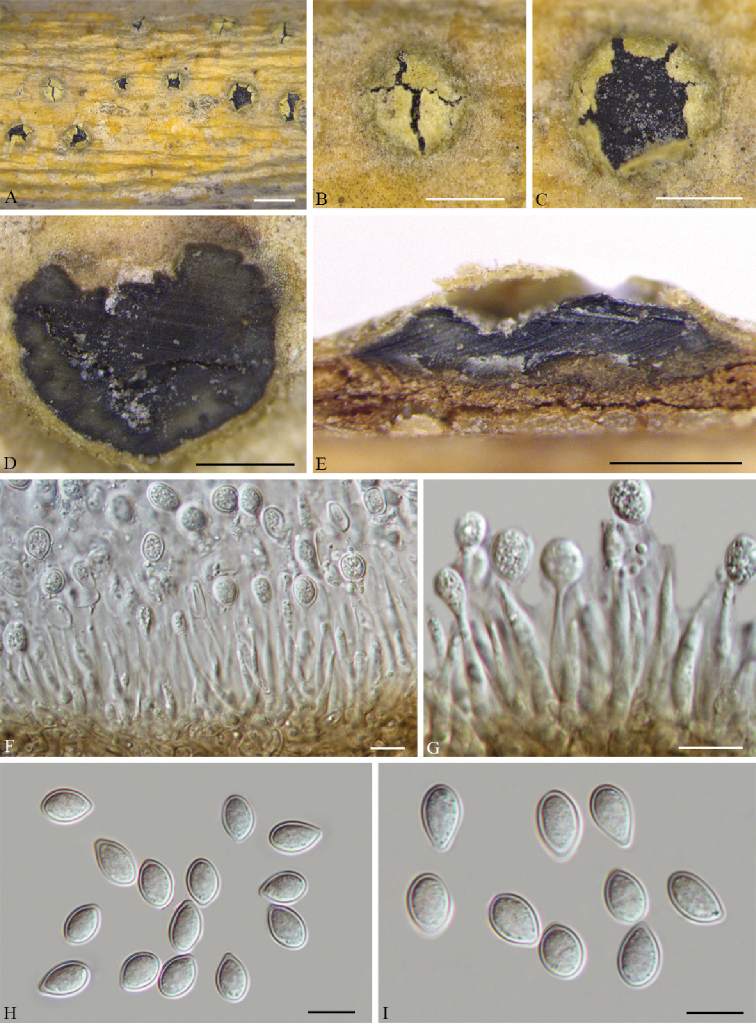
*Plagiostoma populinum* on *Populus tomentosa* (BJFC-S1724) **A–C** habit of conidiomata on twigs **D** transverse section through conidiomata **E** longitudinal section through conidiomata **F, G** conidiogenous cells attached with conidia **H, I** condia. Scale bars: 2 mm (**A**); 1 mm (**B, C**); 500 μm (**D, E**); 10 μm (**F–I**).

#### Specimens examined.

China. Beijing: Haidian district, 40°31'55"N, 116°20'24"E, on branches of *Populus tomentosa*, 12 November 2017, *N. Jiang* (BJFC-S1724; living culture: CFCC 53016 and CFCC 53017).

#### Notes.

*Plagiostoma populinum* is a common plant pathogenic fungus causing poplar canker in China. The current identification follows previous descriptions and records (Butin 1958). In the present study, two isolates (CFCC 53016 and CFCC 53017) from symptomatic branches of *Populus tomentosa* were congruent with *P. populinum* based on morphology and DNA sequences data (Fig. 3). We therefore describe *P. populinum* as a known species for this clade.

## Discussion

In this study, three gnomoniaceous species were identified based on morphological and molecular phylogenetic analyses. As a result, *Cryphognomonia* typified with *C. pini* is proposed as a new genus in Gnomoniaceae for its distinct phylogenic position and distinctive sexual morphs. Also, *Gnomoniopsis xunwuensis* strains were successfully isolated from leaf spot of *Castanopsis fissa*, and were identified as a new species in *Gnomoniopsis*, which was typified by *Gnomoniopsis chamaemori* having pycnidia with hyaline, oval, one-celled conidia (Walker et al. 2010).

The type species of *Cryphognomonia*, *C. pini*, is unique through its developed pseudostromata and ascospores with distinct hyaline sheath. In the molecular phylogeny, *C. pini* is closely related to species of *F. rhoigena*. *Flavignomonia rhoigena* is characterized by the formation of synnemata and no sexual morph is known for this species (Jiang et al. 2019). However, *C. pini* can be easily distinguished from *F. rhoigena* based on ITS, LSU, *tef1* and *rpb2* loci. Therefore, the unique morphology in combination with an isolated phylogenetic position within Gnomoniaceae warrant the establishment of a new genus.

Most species of *Gnomoniopsis* show host preference or potentially limited host specificity to genera in the Fagaceae, Onagraceae and Rosaceae (Sogonov et al. 2008). In the present study, isolates were collected from leaf spot of *Castanopsis fissa*, and described as a novel pathogen depending on its asexual state, *G. xunwuensis*. Four taxa, *G. clavulata*, *G. daii*, *G. paraclavulata*, and *G. smithogilvyi*, have been found on Fagaceae host plants. However, *Gnomoniopsis xunwuensis* can be easily distinguished from the four species in conidial size (16.5–20 × 4–5.5 µm in *G. xunwuensis* vs. 5.0–8.0 × 2.0–4.0 µm in *G. clavulata* vs. 5.0–8.0 × 2.0–3.5 µm in *G. daii* vs. 6.0–9.5 × 2.0–3.5 µm in *G. paraclavulata* vs. 4.9–9.8 × 2.9–4.9 µm in *G. smithogilvyi*), as well as supported by molecular data (Walker et al. 2010; Crous et al. 2012; Visentin et al. 2012).

*Plagiostoma populinum* is regarded as the pathogen responsible for poplar canker. Butin (1958) presented a full description with illustrations of this species as *Cryptodiaporthe populea*. Mejía et al. (2011a) treated *C. populea* as a synonym of *P. populinum* based on analyses of cultural and DNA sequence data. In this paper, *P. populinum* forms a highly supported monophyletic group (Fig. 3) characterized by having conidia with obvious hyaline sheath. It is the first time that we have been able to provide detailed morphological diagrams in China.

## Supplementary Material

XML Treatment for Cryphognomonia

XML Treatment for Cryphognomonia pini

XML Treatment for Gnomoniopsis xunwuensis

XML Treatment for Plagiostoma populinum
